# Rapid encapsulation of true ferns and arborane/fernane compounds fossilised in siderite concretions supports analytical distinction of plant fossils

**DOI:** 10.1038/s41598-023-47009-8

**Published:** 2023-11-13

**Authors:** Madison Tripp, Lorenz Schwark, Jochen J. Brocks, Paul Mayer, Jessica H. Whiteside, William Rickard, Paul. F. Greenwood, Kliti Grice

**Affiliations:** 1 Western Australian Organic and Isotope Geochemistry Centre, The Institute for Geoscience Research, School of Earth and Planetary Sciences, Curtin University, Kent Street, Bentley, WA 6102 Australia; 2 Organic Geochemistry Unit, Institute of Geoscience, Christian-Albrechts-University, 24118 Kiel, Germany; 3 Research School of Earth Sciences, The Australian National University, Canberra, ACT 2601 Australia; 4 The Field Museum, 1400 S Lake Shore Dr., Chicago, IL 60605 USA; 5 Department of Earth and Environmental Sciences, San Diego State University, San Diego, CA 92182 USA; 6 John de Laeter Centre, Curtin University, Kent Street, Bentley, WA 6102 Australia

**Keywords:** Palaeoecology, Geochemistry

## Abstract

Fossilised true ferns (*Pecopteris* sp.) preserved in siderite concretions from the Mazon Creek Lagerstätte (Illinois) presented a unique opportunity to characterise the organic signatures of these late Carboniferous plants. Localised analyses of true fern fossils showed several highly abundant phytohopanoids and fernane/arborane derived aromatic products, which were present only negligibly within their siderite matrix, as well as from other types of fossilised plants. These terpenoids had been recognised in some extant ferns, but scarcely in sedimentary organic matter and their exact source remained ambiguous. The present fossil biomarker data confirms an ancient true fern origin. Furthermore, the excellent concretion preservation of a series of related terpenoid products provided a rare insight into their diagenetic formation. The benign properties of carbonate concretions could be exploited further for biomarker evidence of other fossilised organisms, with one important caveat being that biomarker signals attributed to isolated fossils be significantly distinct from background organic matter pervading the concretion matrix. For instance, hydrocarbon profiles of seed ferns (pteridosperms) and articulates (horsetails) also preserved in Mazon Creek concretions were indistinguishable from separate analysis of their concretion matrix, preventing biomarker recognition.

## Introduction

Rapid encapsulation of organic matter (OM) from the cellular remains of extant organisms in carbonate concretions prior to significant diagenetic alteration can aid the preservation of biomarkers and biomolecules in deep time^1^. The concretion preservation of plant fossils may therefore be represented by biomarkers, as well as macrofossils (often permineralised) or microscopically identifiable spores and pollen. The biomarker record of plant fossils has largely been limited to the ‘selective preservation’ of highly stable biopolymers of cuticles, dominantly the polyester cutin or the polymeric hydrocarbon cutan^2–5^, but also resins and occasionally highly specific terpenoids^6,7^. Plant species devoid of resistant macromolecules may be under-represented in the palaeobotanical record^8^. Resins and waxes are more abundant in sedimentary records than plant terpenoids, but the high structural diversity of the latter offer significant palaeobotanical diagnostic potential. Established terpenoid biomarkers, which have retained a structural resemblance to the biochemicals of modern plants—even after minor diagenetic modification (e.g., loss of functional groups or saturation of double bonds)—present an important opportunity to investigate ancient source-biomarker relationships. The palaeobotanical value of terpenoid biomarkers will also be enhanced with a better understanding of diagenetic characteristics of specific preservation processes (e.g., concretion formation).

Plant biomarkers can help to illuminate long term evolutionary trends (e.g., radiation or decline of specific plant groups/species), which are intimately associated with the palaeoenvironmental dynamics of Earth, including large-scale events such as mass extinctions^9–11^. Land plant biomarkers have been widely reported in petroleum and sedimentary OM since the Late–Middle Devonian (385 to 359 Ma), whereby these biomarkers often predated the first occurrence of morphologically preserved plant fossils^12^. Common plant biomarkers include gymnosperm resin derived phyllocladane, isopimarane and simonellite^13–15^, and the commonly angiosperm-associated oleanoids or lupanoids^16–18^—useful age diagnostic markers of flowering plants^19^. Hopanoids are typically interpreted as a bacterial signal in sedimentary OM^20,21^, but certain structures are indicative of a terrestrial origin, including some ferns. Diplopterol (**I**), diploptene (**II**) and fernenes (**III, IV, V**) were identified in modern ferns^22–26^, although diplopterol (**I**) and diploptene (**II**) have since also been associated with bacteria^20,21^ (see Supplementary Fig. S1 for chemical structures).

Mazon Creek is a Pennsylvanian (late Carboniferous; 306–311 Ma) fossil Lagerstätten, renowned for its unique assemblage of soft tissue fossils found in siderite (iron carbonate) concretions. The concretions preserve an abundance of ancient organisms from a range of localised habitats, encompassing both terrestrial swamps and marine environments^27^. Carboniferous Mazon Creek was a swampy forest near the palaeo-coast^27–30^ and represents a brackish-marine environment^27^. The siderite nodules are found within the Francis Creek Shale, overlying the Colchester Coal (no. 2) Member^31,32^. Carbonate concretion formation occurs as a product of microbial respiration of organic matter and depends on the local environmental influences. Siderite concretions such as those at Mazon Creek are driven by methanogenesis due to limited dissolved sulfate in a freshwater environment^33––35^. The preservation of soft tissue fossils is attributed to the early formation of siderite, encapsulating the organism prior to extensive decay.

Here, we demonstrate the correlation of terpenoid biomarkers with ancient true ferns that have been sequestered and exquisitely preserved within siderite concretions of the Late Carboniferous Mazon Creek Lagerstätte. Specifically, the intimate association of true fern fossils and a suite of saturated phytohopanoid and aromatised terpenoid diagenetic biomarker products supports a direct source from the original true fern material and excludes the probability of other sources.

## Methods

### Sample preparation and extraction

**Figure 1:**
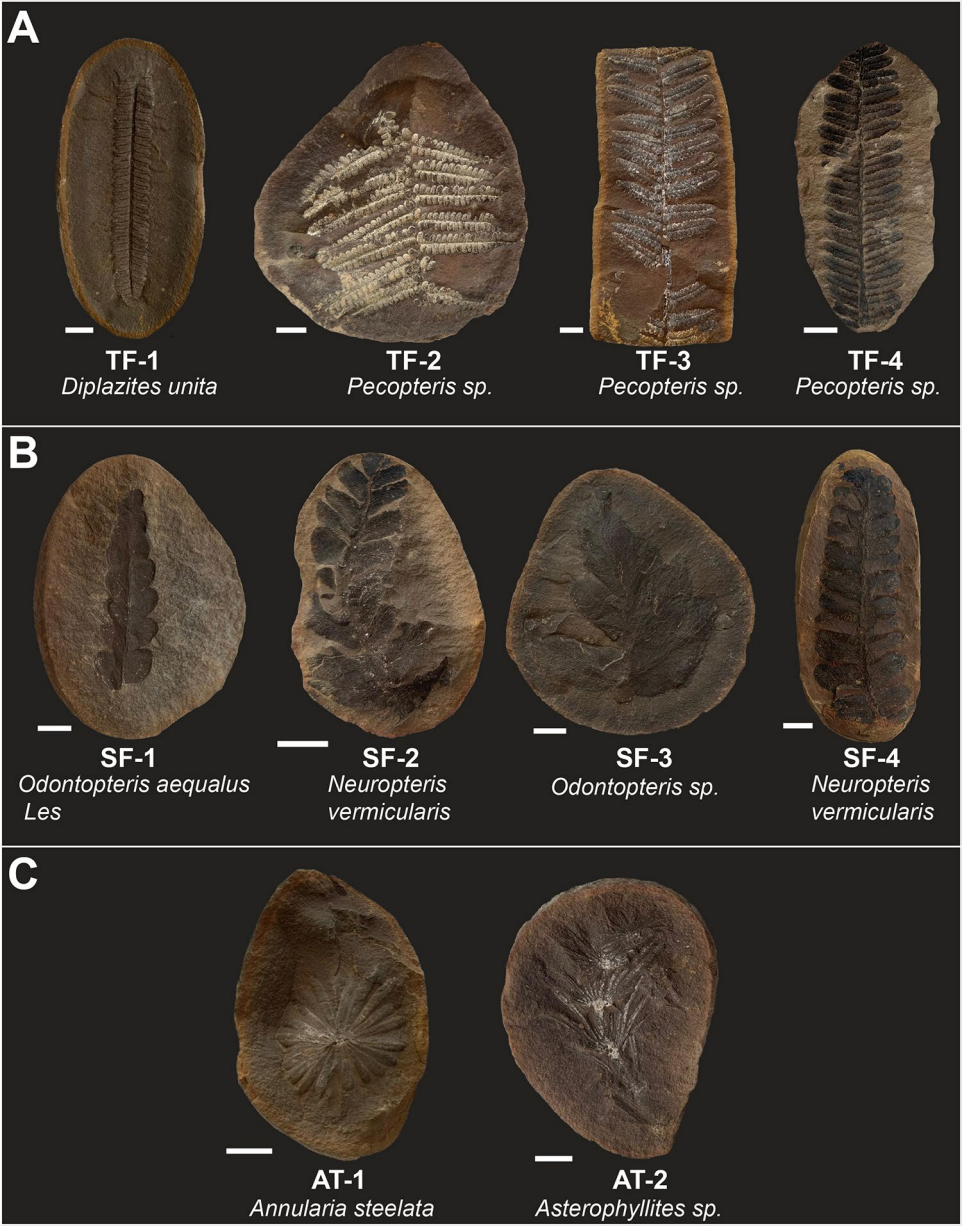
Samples used for analysis include some of the more common species found at Mazon Creek. Fossils range from approximately 3 to 13 cm in length. (**A**) True ferns. These are comprised of *Pecopteris* and *Pecopteris*-type species (Pteridophyta). Leaves are preserved in fine detail, remineralised by iron carbonate and in some cases mineralised by a white mineral (probable clay, based on observation and previous descriptions of Mazon Creek fossils^27,66^. TF-1: FMNH PP39376; TF-2: FMNH PP1476; TF-3: FMNH Uncatalogued; TF-4: FMNH P18752. (**B**) Seed ferns. These include specimens of *Odontopteris* and *Neuropteris* which are preserved as thick dark layers with a ‘waxy’ appearance. SF-1: FMNH P31070; SF-2: FMNH PP26465; SF-3: FMNH PP3743; SF-4: FMNH Uncatalogued. (**C**) Articulates. Preservation style very similar to that of true ferns, with some white mineral (probable clay). AT-1: FMNH PP16930; AT-2: FMNH PP20624. White bar represents 1 cm relative to each individual sample.

Samples were obtained from the Field Museum (Chicago, USA) and consist of a variety of plant fossil species preserved in siderite concretions (Fig. 1). One half of each fossil concretion was cut into separate sections using a handheld Dremel rotary tool with a diamond blade (previously cleaned by sonicating in a mixture of dichloromethane (DCM) and methanol (MeOH) (9:1 *v/v*) for 15 min intervals), separating the fossil leaf (referred to as ‘fossil’) from the bulk of the surrounding matrix (‘outer matrix’). Where possible, the matrix was divided into two regions—immediately surrounding the fossil and the outer rim, referred to as the ‘inner matrix’ and ‘outer matrix’, respectively. The different sections of each concretion were washed by repeated sonication in 15 min intervals to remove a proportion of external contamination, in a mixture of DCM:MeOH (9:1 *v/v*), before being ground using pre-annealed (500 °C for 2 h) ceramic pestles and mortars. In between sample treatments, pre-annealed sand was ground to ensure the mortars were thoroughly cleaned. Ground sample material was Soxhlet-extracted in individual pre-extracted cellulose thimbles for 72 h. A procedural blank consisting of a pre-extracted thimble was run alongside each extraction. Activated copper turnings (4 M HCl) were added to each extract to remove elemental sulfur. Small scale column chromatography (5 cm × 0.5 cm i.d.) using silica gel activated at 160 °C for 24 h was used to separate the total lipid extracts into aliphatic (4 mL *n*-hexane), aromatic (4 mL *n*-hexane:DCM (9:1 *v/v*)), porphyrin (4 mL *n*-hexane:DCM (7:3 *v/v*)) and polar (4 mL DCM:MeOH (1:1 *v/v*)) fractions for analysis.

### Total organic carbon

The rock samples were ground to a fine powder and digested in hydrochloric acid (HCl) acid to remove the carbonate minerals. The remaining residues were analysed using a LECO Carbon–Sulfur Analyser (CS-230). The CO_2_ produced was measured with an infra-red detector, and values calculated according to standard calibration.

### Gas chromatography–mass spectrometry

Full scan gas chromatography–mass spectrometry analysis (GC–MS) was performed on the aliphatic fractions using an Agilent 7890B GC with a DB-1MS UI capillary column (J&W Scientific, 60 m, 0.25 mm i.d., 0.25 μm film thickness) coupled to an Agilent 5977B MSD. Aromatic fractions were analysed on an Agilent 6890N GC with a DB-5MS UI capillary column (J&W Scientific, 60 m, 0.25 mm i.d., 0.25 μm film thickness) coupled to an Agilent 5975B MSD. The GC oven was ramped from 40 to 325 °C at a rate of 3 °C/min with initial and final hold times of 1 min and 30 min, respectively.

Saturated steroids and hopanoids for samples TF-2, TF-4 (true ferns), SF-1, and SF-2 (seed ferns) were identified by additional GC–MS analyses of the aliphatic fraction, on an Agilent 6890 GC with a DB-5 capillary column (J&W Scientific, 60 m, 0.25 mm i.d., 0.25 μm film thickness) coupled to a Micromass Autospec Premier double sector MS (Waters Corporation, Milford, MA, USA). Helium was used as the carrier gas at a constant flow of 1 mL/min. Samples were injected in splitless mode into a Gerstel PTV injector at 60 °C (held for 0.1 min) and heated at 260 °C/min to 300 °C. The MS source was operated at 260 °C in EI mode at 70 eV ionization energy and 8000 V acceleration voltage. All samples were injected in *n*-hexane to avoid deterioration of chromatographic signals by FeCl_2_ build-up in the MS ion source through use of halogenated solvents^36^. The GC oven was programmed from 60 °C (held for 4 min) to 315 °C at 4 °C/min, with a total run time of 100 min. Saturated steranes and hopanes were identified using metastable reaction monitoring (MRM) in M^•+^  → 217 and M^•+^  → 191 transitions, respectively. All ratios and abundance proportions are reported uncorrected for differences in MS-response.

Saturated steroids and hopanoids in aliphatic fraction of samples TF-1, TF-3, SF-3, SF-4, AT-1, and AT-2 were identified using gas chromatography triple quadrupole mass spectrometry (GC-MSMS) using an Agilent 7890A GC with a DB-5MS UI capillary column (J&W Scientific, 60 m, 0.25 mm i.d., 0.25 μm film thickness) coupled to a 7000A triple quadrupole MS. The GC oven was ramped from 60 to 220 °C at a rate of 8 °C/min with an initial hold time of 2 min, then ramped to 320 °C at a rate of 2 °C/min with a final hold time of 28 min. Steranes and hopanes were identified using the M^⋅+^  → 217 and M^⋅+^  → 191 transitions, respectively.

## Results

A range of fossil fern specimens that have been exceptionally preserved within siderite concretions from the Carboniferous Mazon Creek (Illinois) were analysed, including true ferns (Fig. 1A), seed ferns (Fig. 1B) and articulates (Fig. 1C). ‘True ferns’ are here defined as ferns of the *Pecopteris* type, as well as one specimen of *Diplazites unita,* which like *Pecopteris*, is of the Pteridophyta division; while ‘seed ferns’ are referring to a range of species of Pteridosperms. Total organic carbon content of isolated concretion material ranged from 0.23 to 0.44 wt%. Biomarker distributions were investigated by gas chromatography-mass spectrometry (GC–MS). The molecular analysis of each fossil was accompanied by corresponding analysis of the surrounding matrix region to establish the integrity of the fossil data. Concretions with sufficient matrix volume were further separated into inner and outer matrix regions. Distinct differences in molecular composition were evident between the fossil and matrix regions of true fern specimens (TF-1 to TF-4) (Fig. 1A), while the hydrocarbon composition of the concretions containing seed ferns and articulates were largely homogeneous.

**Figure 2:**
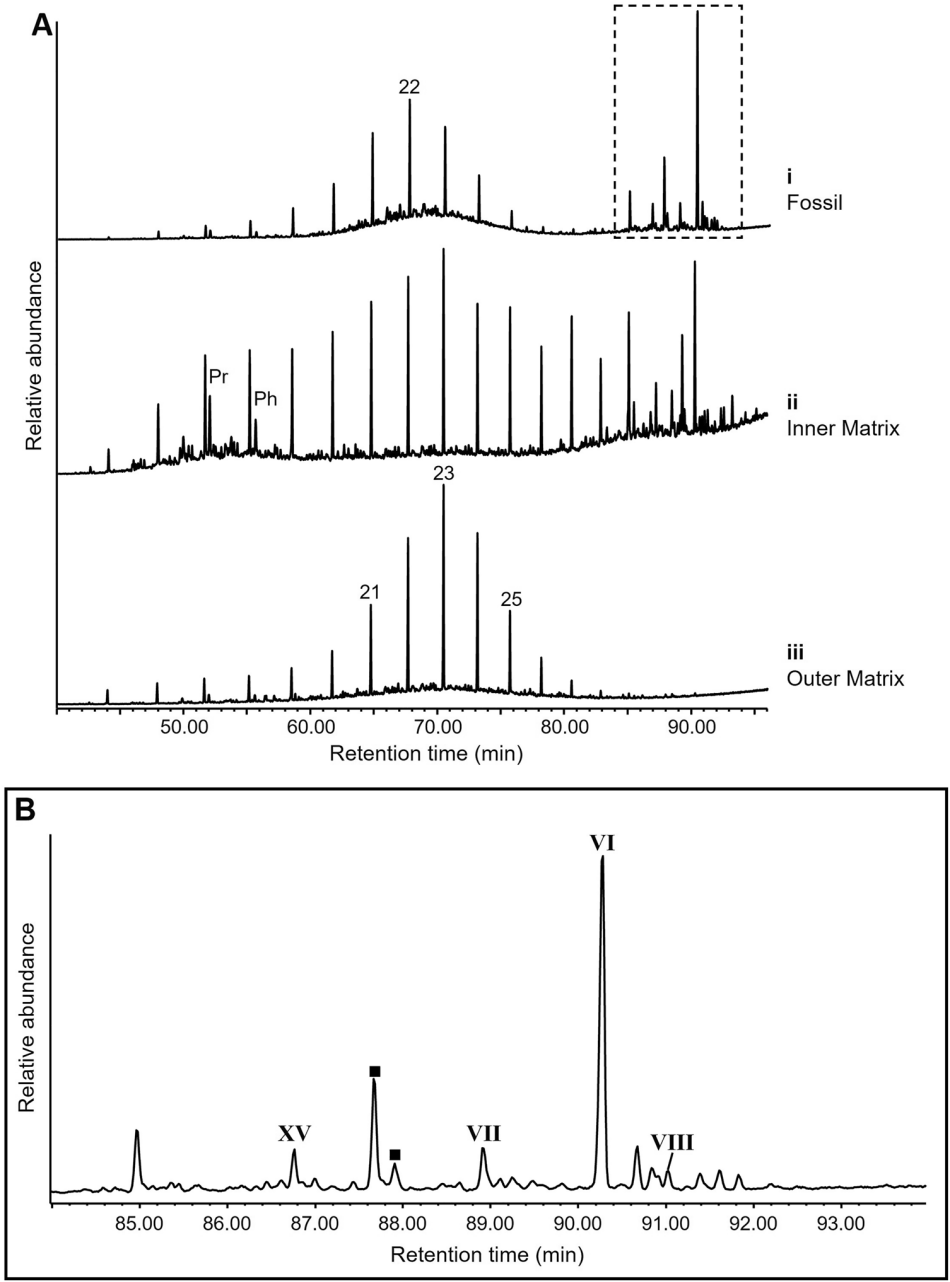
(**A**) GC–MS total ion chromatograms of aliphatic fractions of true fern sample TF-1 (*Diplazites unita*) (**i**) fossil, (**ii**) inner matrix, and (**iii**) outer matrix. Numbers represent carbon number of straight-chain *n*-alkanes; *Pr* pristane, *Ph* phytane. (**B**) Triterpenoid region of aliphatic fraction of TF-1 fossil. Peaks are labelled using roman numerals according to structure numbers in Fig. S1. C_30_ hopenes which have been approximately assigned based on their mass spectra are indicated by black squares (mass spectra are shown in Fig. S3A).

**Figure 3:**
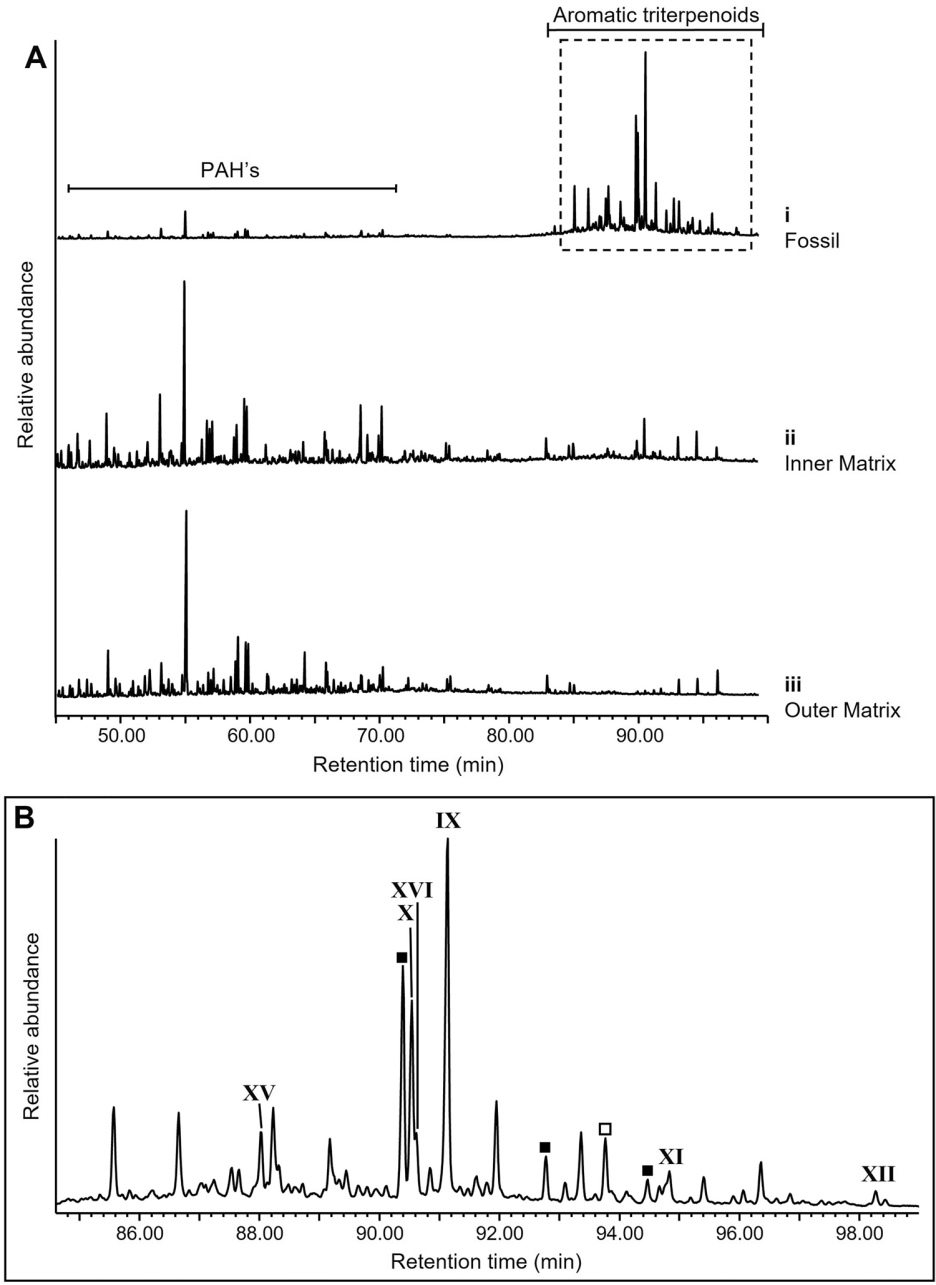
(**A**) GC–MS total ion chromatograms of aromatic fractions of true fern sample TF-1 (*Diplazites unita*) (**i**) fossil, (**ii**) inner matrix, and (**iii**) outer matrix. (**B**) Triterpenoid region of aromatic fraction of TF-1 fossil. Peaks labelled using roman numerals according to structure numbers in Fig. S1. Compounds were identified by comparison of their mass spectra with those reported in the literature^7,37,40,41,48^ (Fig. S3B). Other unidentified peaks had mass spectra that suggested a relationship to a DAPH (**XI**, **XII**) structure, denoted in the figure by squares. Filled squares represent probable C_28_ diaromatic compounds and the empty square represents a probable C_29_ diaromatic compound (examples with relevant retention time shown in Fig. S3C).

The true fern specimens (TF-1–TF-4) contained abundant C_30_ saturated triterpenoids, predominantly C_30_ αβ hopane (**VI**), accompanied by C_30_ diahopane (**VII**), C_30_ βα hopane (**VIII**) and C_30_ hopenes (e.g., Fig. 2; Supplementary Fig. S2). Highest C_30_ hopanoid abundances were localised to the fossil regions of samples TF-1–TF-3, reflected by C_29_/C_30_ αβ hopane ratios ranging from 0.01 to 0.06; cf. 0.20 to 0.57 in inner matrices and 0.50 to 0.92 in outer matrices of all four true fern specimens (Supplementary Table S1, Supplementary Fig. S2). The high localised hopane signal of the true fern fossils TF-1–TF-3 was also reflected by regular steranes/hopanes ratios of 0.01 to 0.09 (Supplementary Table S1); cf. 0.12 to 0.25 in inner matrices and 0.35 to 0.56 in outer matrices. The C_29_/C_30_ αβ hopane ratio of 0.18 in the fossil region of TF-4, and regular steranes/hopanes of 0.15, also supported a C_30_ hopanoid predominance, although less significant than in TF-1–TF-3. Negligible evidence of extended hopanoids (i.e., C_31_–C_35_ hopanoids), which form from C_35_ bacteriohopanepolyol specific to heterotrophic bacteria or cyanobacteria^37–39^, associated with true fern fossils excluded a significant hopane contribution from bacterial sources. Aromatic fractions of the true fern fossil samples (TF-1–TF-3) showed a distinctive distribution of predominantly C_30_ aromatic triterpenoid compounds, analogous to the saturated hydrocarbon distributions (Fig. 3, Supplementary Fig. S3). These products were present only in trace abundances in the matrix regions, which were instead dominated by low molecular weight polycyclic aromatic hydrocarbons (e.g., parent and alkyl naphthalenes and phenanthrenes). The aromatic triterpenoids in the true fern fossils included 5-methyl-10(4-methylpentyl)des-A-25-*nor*arbora(ferna)-5,7,9-triene (Monoaromatic Tetracyclic Hydrocarbon = MATH) (**IX**), 25-*nor*arbora(ferna)-5,7,9-triene (Monoaromatic Pentacyclic Hydrocarbon = MAPH) (**X**), 24,25-*dinor*arbora(ferna)-1,3,5,7,9-pentaene (Diaromatic Pentacyclic Hydrocarbon = DAPH-1) (**XI**) and *iso-*25-*nor*arbora(ferna)-1,3,5,7,9-pentaene (DAPH-2) (**XII**) (Fig. 3, Supplementary Fig. S3B), which are known to be derived from arborane/fernane precursor skeletons, including fern-9(11)-en-3β-ol (**XIII**) and arbor-9(11)-en-3β-ol (**XIV**)^37, 40^. Several other aromatic products showed very similar mass spectra to the aromatised fernane/arborane series (Fig. 3, Supplementary Fig. S3C). Also present were C_30_ homologs of the D-ring monoaromatic 8,14-secohopanoid series (**XV**) as identified by Hussler et al.^41^, and C_30_ indenyldrimanes (**XVI**) first identified by Killops^42^, characterised by selected *m/z* 365 (Supplementary Fig. S4A) and 363 (Supplementary Fig. S4B) chromatograms, respectively. Desmethyl D-ring monoaromatic 8,14-secohopanes (**XVII**) are identified in the *m/z* 351 ion chromatogram of sample TF-1 (cf. literature data^43^) (Supplementary Fig. S4C).

In true fern fossils, the C_30_ carbon number bias (or derivation from C_30_ precursor biomolecules) of aromatic triterpenoids mirrors the C_30_ hopanoid prevalence of their saturated hydrocarbon distribution, reflecting a probable relationship. Furthermore, there was a consistent absence of bacterial sourced markers including benzohopanes and the extended hopanes^44^ in the saturated hydrocarbon distributions. The C_30_ hopanoid predominance of TF-4 was less than the other specimens (Supplementary Table S1) and it lacked abundant aromatic triterpenoids, which may be attributed to a lower preservation quality.

**Figure 4:**
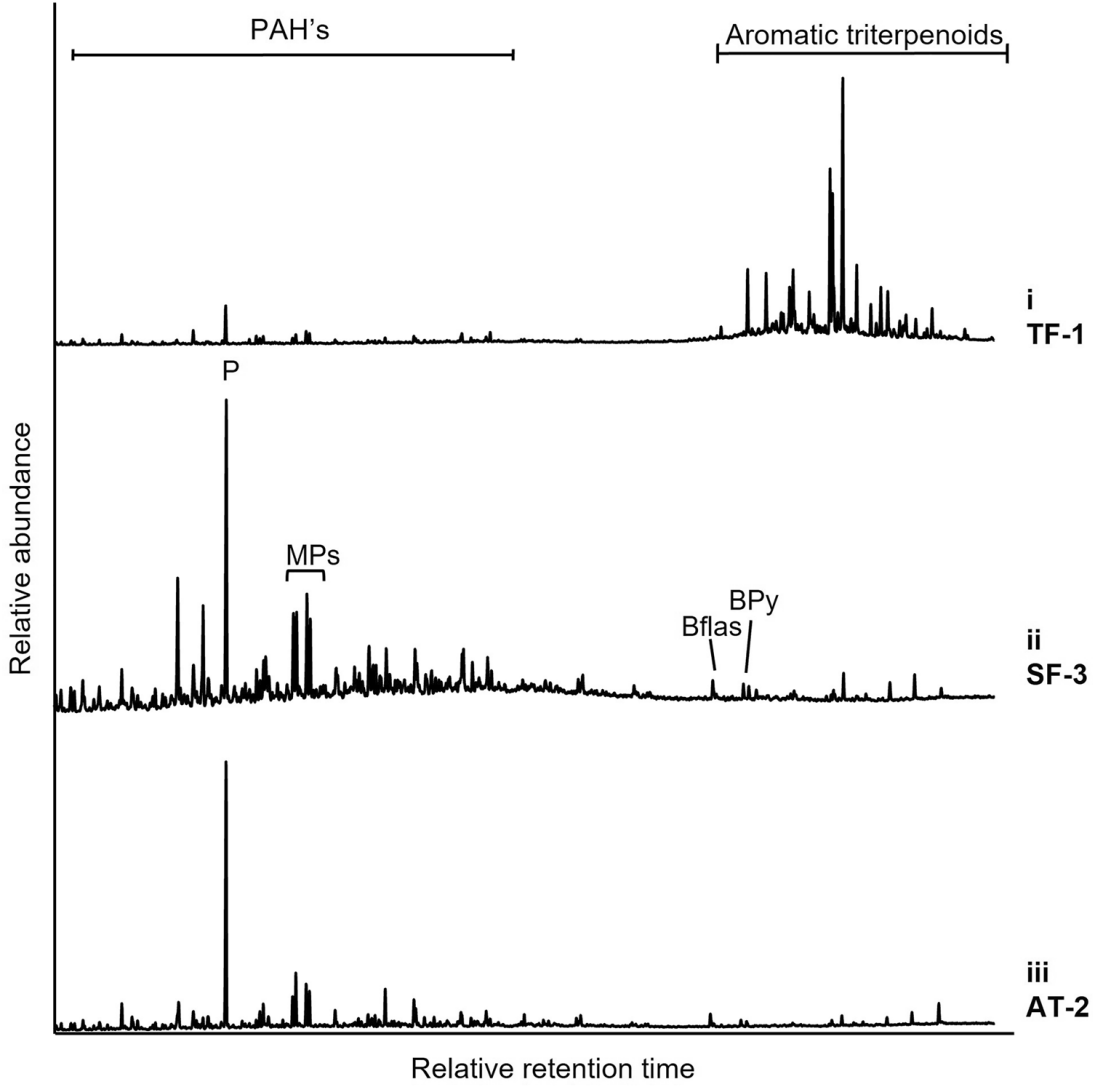
GC–MS total ion chromatograms of aromatic fractions of: **(i)** *Diplazites unita* TF-1; **(ii)** *Odontopteris* sp. SF-3; **(iii)** *Asterophyllites* sp. AT-2. *PAH’s* polycyclic aromatic hydrocarbons, *P* phenanthrene, *MPs* methylphenanthrenes, *BFlas* benzofluoranthenes, *BPy* benzopyrenes.

In contrast to the true ferns, the aromatic (Fig. 4) and aliphatic (Supplementary Fig. S5) terpenoid compositions of the seed fern (Fig. 1B) and articulate plant (Fig. 1C) fossils showed little difference to that of their adjacent matrix (see biomarker ratios in Supplementary Table S1), which precludes the identification of any biomarkers of these plant sources. Trace amounts of aromatised fernane/arborane hydrocarbons were detected in a few seed fern samples (Fig. 4), but they were similarly observed in their outer matrix. Most of these plants contained C_29_ and C_30_ αβ hopanes of similar abundances to one another, and bacterially derived extended hopanoids up to C_33_ (e.g., Supplementary Fig. S5).

## Discussion

The abundant suite of C_30_ hopanoids and aromatic fernanes/arboranes detected in the Carboniferous true fern fossils, in high abundances compared to their concretion matrices, identifies a unique biomarker-source relationship not shared by seed fern or articulate plant fossils. Notably, the distribution of C_30_ triterpenoid compounds in the true ferns is comparable to phytohopanoid precursors, which have been observed in extant plants, including ferns. Compounds of the hopene, neohopene, fernene, and oleanene groups, among others, were identified in extant species of Japanese ferns and fungi^22,23,45^ but have also been identified in angiosperm plants^46,47^.

Extended series of D-ring monoaromatic 8,14-secohopanoids have previously been associated with bacteriohopanols^48^, but the C_30_ exclusivity of these products (in association with other related hopanoids) in the fossilised true fern implies diplopterol or diploptene derivation^49^. Aromatic products sharing a basic fernane/arborane structural skeleton, including MATH (**IX**), MAPH (**X**) and DAPHs (**XI** and **XII**)^7,37,40^, are usually interpreted as derived from fern-9(11)-en-3β-ol (**XIII**) or arbor-9(11)-en-3β-ol (isoarborinol) (**XIV**), although their specific stereoisomeric configuration cannot be determined from mass spectral data alone. Isoarborinol, which bears the same hopanoid methylation pattern as fernanes, was previously considered to be strictly related to flowering plants, given the ubiquity of C-3 oxygenated triterpenes in flowering plants and their absence in ferns^25^. However, aromatised isoarborinol derivatives have been detected in sediments of Permian and Triassic age^37,40,50^, which predate the evolution of angiosperms^51^. Their sedimentary source is therefore often ambiguous. Only a small number of studies have associated aromatised fernane/arborane products of ancient sedimentary samples with plant inputs. Compounds MATH (**IX**), MAPH (**X**) and DAPH-1 (**XI**) were first isolated and characterised by Hauke et al.^40^ in Eocene shales and have also been detected in the Permian Kupferschiefer^40,52,53^. Hauke et al.^37^ detected B-ring aromatised triterpenoids (including MAPH **X**) related to fernane/arborane structures in a Triassic black shale and Jurassic laminated bituminous limestone. The E-ring isopropyl group of MATH (**IX**) and several other shale/bitumen products correlates with fernene, fernen-3β-ol or isoarborinol structures of plant precursors, rather than bacterial hopanols^37^. Vliex et al.^7^ associated MATH (**IX**), MAPH (**X**) and DAPH-1 (**XI**) with early gymnosperm plants, including Pteridospermales and Coniferophytes, which were observed in increasing abundance from Westphalian D of Upper Carboniferous and Lower Permian strata. Paull et al.^54^ attributed a range of fernenes and fernanes in Triassic mudstones and coals, to the *Dicroidium* seed fern fronds observed through the sedimentary sequence. More recently, Auras et al.^6^ identified MATH, MAPH and DAPH-1 in cordaitean macrofossils from the Late Carboniferous and noted that they were absent in *Pecopteris* and *Calamites*.

The present data from the Mazon Creek plant fossils specifically attributes the phytohopanoid products to a true fern origin, rather than from seed ferns or articulates, with high confidence based on the separate molecular analyses of several integral true fern specimens isolated within siderite concretions. This is a variation from the previous assignment of aromatic fernane/arborane derived compounds to a cordaite source, based on studies of other Carboniferous plant fossils preserved in Pennsylvanian (Westphalian D) coal^6^.

**Figure 5:**
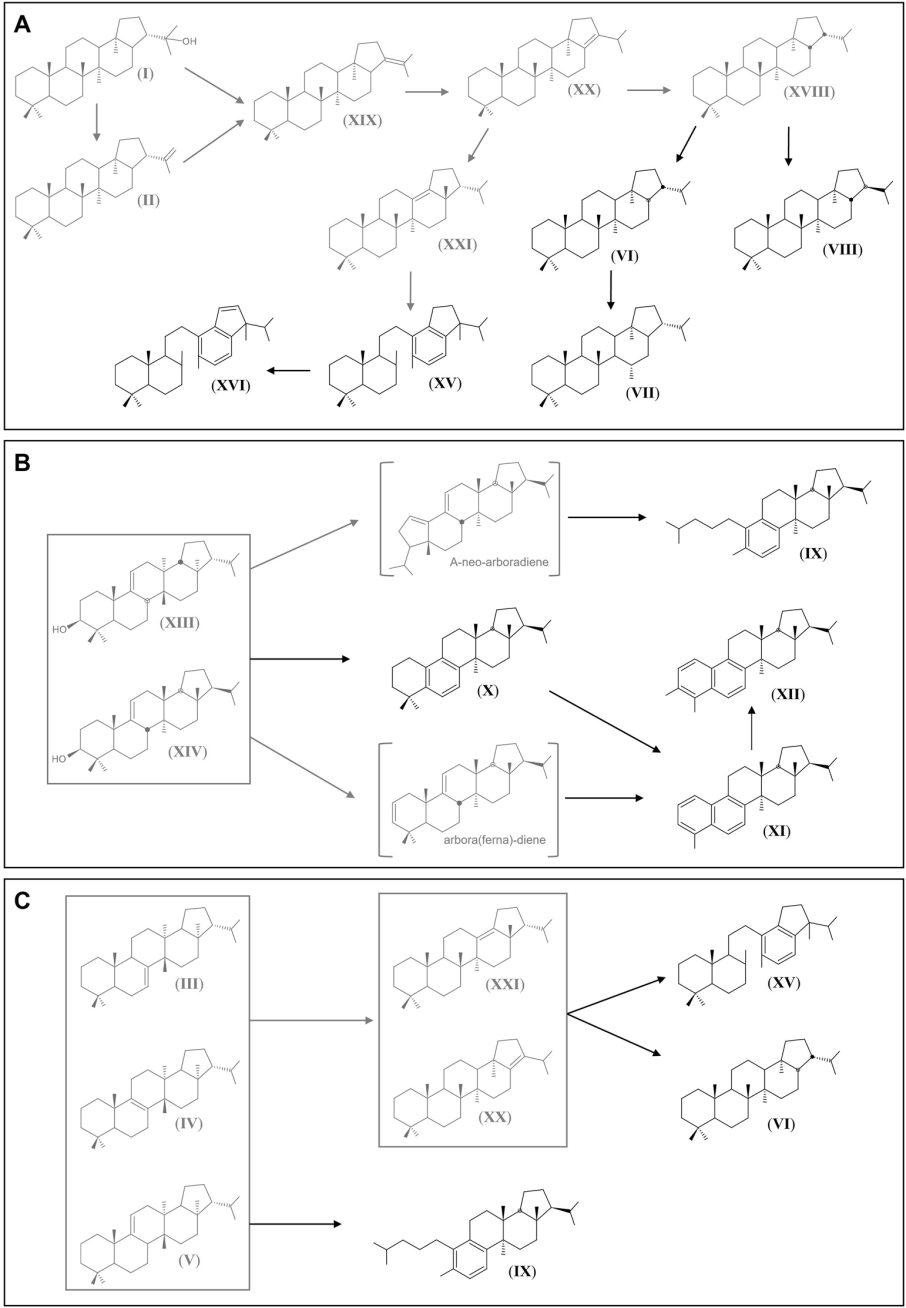
(**A**) Simplified mechanism of formation of C_30_ αβ-hopane (**VI**) and derivatives from diplopterol (**I**) and diploptene (**II**) precursors (structures in grey represent precursors and intermediates, structures in black are identified in samples from this study). (**B**) Proposed mechanism by Vliex et al.^7^ of formation of compounds MATH (**IX**), MAPH (**X**), DAPH-1 (**XI**) and DAPH-2 (**XII**), from fern-9(11)-en-3β-ol (**XIII**) and arbor-9(11)-en-3β-ol (**XIV**). Derivatives are shown with an arborane isomeric configuration, although both would be possible, for ease of demonstration. (**C**) Simplified mechanism showing possible formation of D-ring monoaromatic 8,14-secohopane (**XV**), hop-17(21)-ene (**XX**) and MATH (**IX**) from the fernene isomers (**III, IV, V**).

The Mazon Creek true fern fossils showed a broad suite of phytohopanoid and fernane/arborane diagenetic products. Structurally related saturated and aromatic compounds inform about the diagenetic rearrangement of precursor biomolecules into more stable biomarkers. C_30_ αβ hopane (**VI**) and C_30_ diahopane (**VII**), which overwhelmingly dominated in true fern fossils may be more thermally stable stereoisomer products of C_30_ ββ-hopane (**XVIII**), which could derive via hop-21-ene (**XIX**) and hop-17(21)-ene (**XX**) intermediates, from the diagenetic rearrangement of diplopterol (**I**) and diploptene (**II**) (Fig. 5A). D-ring monoaromatic 8,14-*seco*hopanoids (**XV**), and derivatives (**XVI** and **XVII**), are probably formed by either microbially or thermally mediated C-ring opening^42,48,55^. With even further aromatisation the D-ring monoaromatic 8,14-*seco*hopanoids (**XV**) can progress to form indenyldrimanes (**XVI**)^42,43,48^ (Fig. 5A). A wider series (C_27_–C_35_) of monoaromatic secohopanoids can be bacterially sourced, formed from C_35_ bacterial hopanoid precursors^56^ at a later stage of diagenesis than benzohopanoid formation due to the high energy demands required for D-ring aromatisation^48^; and C-ring opening to produce tetracyclic terpanes^57,58^. The true fern fossils only showed C_30_ homologs of the D-ring monoaromatic 8,14-secohopanoids (and respective demethylated counterparts), and C_30_ indenyldrimanes are present. The absence of higher carbon members excludes the likelihood of derivation from the extended hopanoids of bacteria. Instead, C_30_ exclusivity implies the monoaromatic 8,14-secohopanoids are microbially rearranged products of phytohopanoid precursors.

B-ring monoaromatic and A/B-ring diaromatic fernane/arborane hydrocarbons are most likely of a fernen-3β-ol/isoarborinol source^37, 40^. Aromatisation at the B-ring could initiate at the oxygenated C-3^37,40^, or the double bond at C-9(11)^7^ (Fig. 5B).

Ageta et al.^49^ determined that fernenes can be rearranged via acid-catalysed reaction under various conditions to form neohop-13(18)-ene (**XXI**) and C_30_ αβ hopane (**VI**) (Fig. 5C). Fernenes could potentially then be rearranged to produce C_30_ hopane (**VI**) and D-ring monoaromatic 8(14)-secohopanoids (**XV**)^7^ according to the reaction depicted in Fig. 5A. Vliex et al.^7^ also suggested that 3-des-oxygenated triterpenoids might similarly rearrange to form arborane/fernane-related aromatic triterpenoids, including transformation of fernene into MATH (**IX**) (proposed mechanism by Vliex et al.^7^; Fig. 5C), in a mechanism similar to that observed to occur in diterpenoids lacking C-3 oxygen functionality^55^. Fernanes or fernenes were not identified in the true fern fossils, supporting that fernane/arborane precursors may preferentially form more thermally stable aromatic derivatives, resulting in their preservation^50^. Aromatisation, which has seemingly influenced to some extent the molecular composition of the fossil true ferns, likely occurred early in the diagenetic history of the sample during concretion growth and formation.

Extant organisms sequestered in isolation as concretion fossils represent a valuable target for biomarker characterisation and molecular evolution studies^1,59–62^. The rapid encapsulation of individual organisms in low permeability carbonate minerals effectively implements a closed chemical system, inhibiting diagenetic decay or microbial invasion and supporting the preservation of cellular remains and molecular biomarkers^63–65^. Presently, true ferns are distinguished from other plant types represented in Mazon Creek concretions based on the exclusive detection of C_30_ phytohopanoids and aromatised fernenes associated with preserved fossils. These biomarkers were largely absent from analysis of the encapsulating concretion—apart from trace amounts in the inner matrix in close proximity to the fossil ferns—demonstrating that fossil-derived organic matter contributed negligibly to the background hydrocarbon distributions of the concretion matrices. Therefore, despite conflicting evidence from previous studies, we are able to assign here a true fern source to the phytohopanoid compounds, which dominate the biomarker distributions of these specimens, including C_30_ αβ hopane (**VI**) and its derivatives, D-ring monoaromatic 8,14-secohopanoid (**XV**) and its derivatives, and the diagenetically aromatised arborane/fernane compounds MATH (**IX**), MAPH (**X**), DAPH-1 (**XI**) and DAPH-2 (**XII**). The results of this study present novel insights into the utility of concretion fossil analyses for identification of biomarkers or ancient organisms. The encouraging assignment of Carboniferous true fern biomarkers provides impetus for similar characterisation of other organisms fossilised in concretion from Mazon Creek or other world-famous fossil sites (e.g., Green River, USA), or in other similarly benign mineral settings (e.g., cherts), with important implications for understanding biomarker records through time and interpreting molecular relationships between ancient and extant species.

### Supplementary Information

Supplementary Information.

## Data Availability

Data can be made available upon request from corresponding authors.
