# Supplementary material for: Rapid encapsulation of true ferns and arborane/fernane compounds fossilised in siderite concretions supports analytical distinction of plant fossils
Source: Sci Rep. 2023 Nov 13;13:19851. doi: 10.1038/s41598-023-47009-8 (PMC10646143; doi:10.1038/s41598-023-47009-8)
Supplement: Supplementary file 1 — Supplementary Information. [file 41598_2023_47009_MOESM1_ESM.pdf]

Supplementary materials

Rapid encapsulation of true ferns and arborane/fernane compounds fossilised in siderite concretions supports analytical distinction of plant fossils

Madison Tripp[1*], Lorenz Schwark[1, 2], Jochen J. Brocks[3], Paul Mayer[4], Jessica H. Whiteside[5], William Rickard[6], Paul. F. Greenwood[1], Kliti Grice[1*]

[1] Western Australian Organic and Isotope Geochemistry Centre, The Institute for Geoscience Research, School of Earth and Planetary Sciences, Curtin University, Kent Street, Bentley, WA 6102, Australia

[2] Organic Geochemistry Unit, Institute of Geoscience, Christian-Albrechts-University, 24118 Kiel, Germany

[3] Research School of Earth Sciences, The Australian National University, Canberra, ACT 2601, Australia

[4] The Field Museum, 1400 S Lake Shore Dr., Chicago, IL 60605, USA

[5] Department of Earth and Environmental Sciences, San Diego State University, CA 92182, USA

[6] John de Laeter Centre, Curtin University, Kent Street, Bentley, WA 6102, Australia

*Corresponding author: Madison.tripp@curtin.edu.au; K.Grice@curtin.edu.au

Table S1. Biomarker distributions in extracted organic matter. All ratios were calculated using area of peaks obtained from GC-MS analysis.

			Reg steranes/ 17α hopanes	C_{29}/C_{30} αβ hopanes	βα/αβ C_{30} hopanes
**True ferns**	*TF-1*	**F**	0.01	0.01	0.06
		**IM**	0.12	0.20	0.16
		**OM**	0.48	0.92	0.19
	TF-2	**F**	0.02	0.02	0.07
		**IM**	0.25	0.57	0.20
		**OM**	0.39	0.80	0.18
	TF-3	**F**	0.09	0.06	0.07
		**OM**	0.56	0.53	0.18
	TF-4	**F**	0.15	0.18	0.11
		**OM**	0.35	0.50	0.14
**Seed ferns**	*SF-1*	**F**	0.19	0.30	0.13
		**IM**	0.11	0.22	0.15
		**OM**	0.46	0.96	0.19
	SF-2	**F**	0.38	0.72	0.16
		**OM**	0.38	0.94	0.22
	SF-3	**F**	0.16	0.66	0.15
		**OM**	0.30	0.71	0.14
	SF-4	**F**	0.19	0.83	0.40
		**OM**	0.40	1.07	0.23
**Articulates**	*AT-1*	**F**	0.21	0.45	0.15
		**OM**	0.43	1.00	0.20
	AT-2	**F**	0.25	0.46	0.14
		**OM**	0.41	0.93	0.24

* C_{29} βα 20*S* coelutes with C_{27} αββ 20*R*; same peak used for both compounds in ratio determinations, may affect values.

Reg steranes/17α hopanes = [Σ(C_{27}-C_{29} steranes)]/[Σ(C_{29}-C_{33} hopanes)]; Regular steranes = C_{27}-C_{29} ααα- and αββ-20(*S*+*R*)-steranes; ααα = 5α(H),14α(H),17α(H); αββ = 5α(H),14β(H),17β(H); 17α hopanes = C_{29} αβ, C_{30} αβ, C_{31}-C_{33} αβ 22(*S*+*R*) (where present); αβ = 17α(H),21β(H). Ts = C_{27} 18α-22,29,30-tris*norneo*hopane; Tm = C_{27} 17α-22,29,30-trisnorhopane.

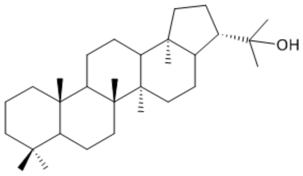

I diplopterol

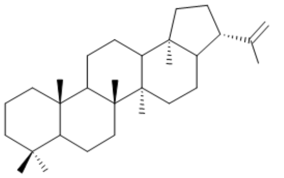

II diploptene

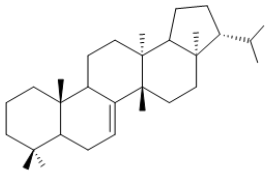

III fern-7-ene

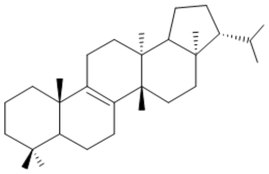

IV fern-8-ene

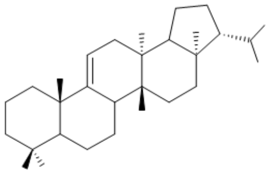

V fern-9(11)-ene

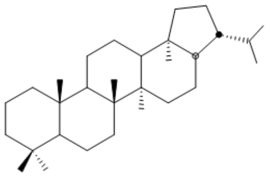

VI C_{30} αβ-hopane

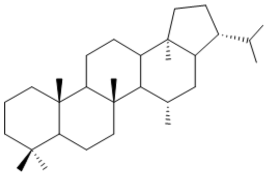

VII C_{30} diahopane

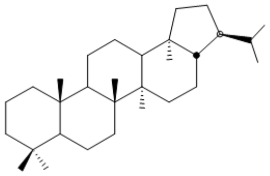

VIII C_{30} βα-hopane

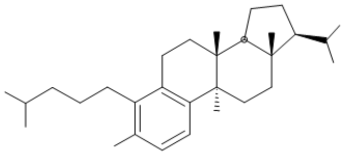

IX 5-methyl-10(4-methylpentyl)-des-*A*-25-norarbora(ferna)-5,7,9-triene

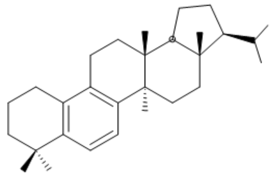

X 25-norabora(ferna)-5,7,9-triene

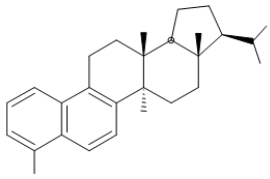

XI 24,25-dinorarbora(ferna)-1,3,5,7,9-pentaene

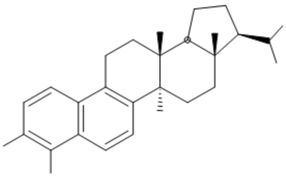

XII *iso*-25-norarbora(ferna)-1,3,5,7,9-pentaene

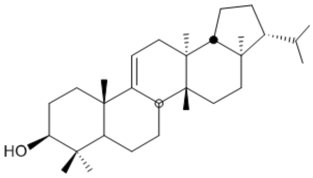

XIII fern-9(11)-en-3β-ol

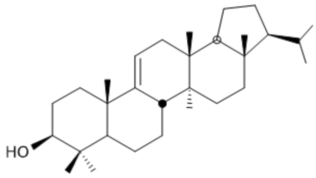

XIV arbor-9(11)-en-3β-ol

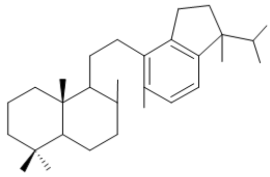

XV D-ring monoaromatic 8,14-secohopanoid

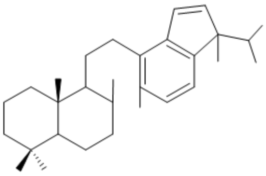

XVI indenyldrimane

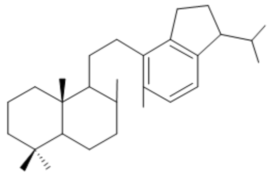

XVII demethylated D-ring monoaromatic 8,14-secohopanoid

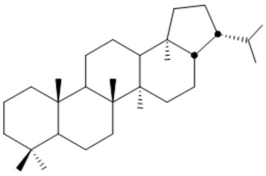

XVIII C_{30} ββ-hopane

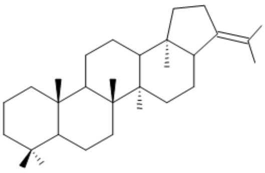

XIX hop-21-ene

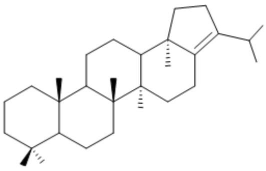

XX hop-17(21)-ene

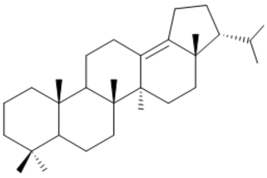

XXI neohop-13(18)-ene

Figure S1. Structures of compounds discussed in text.

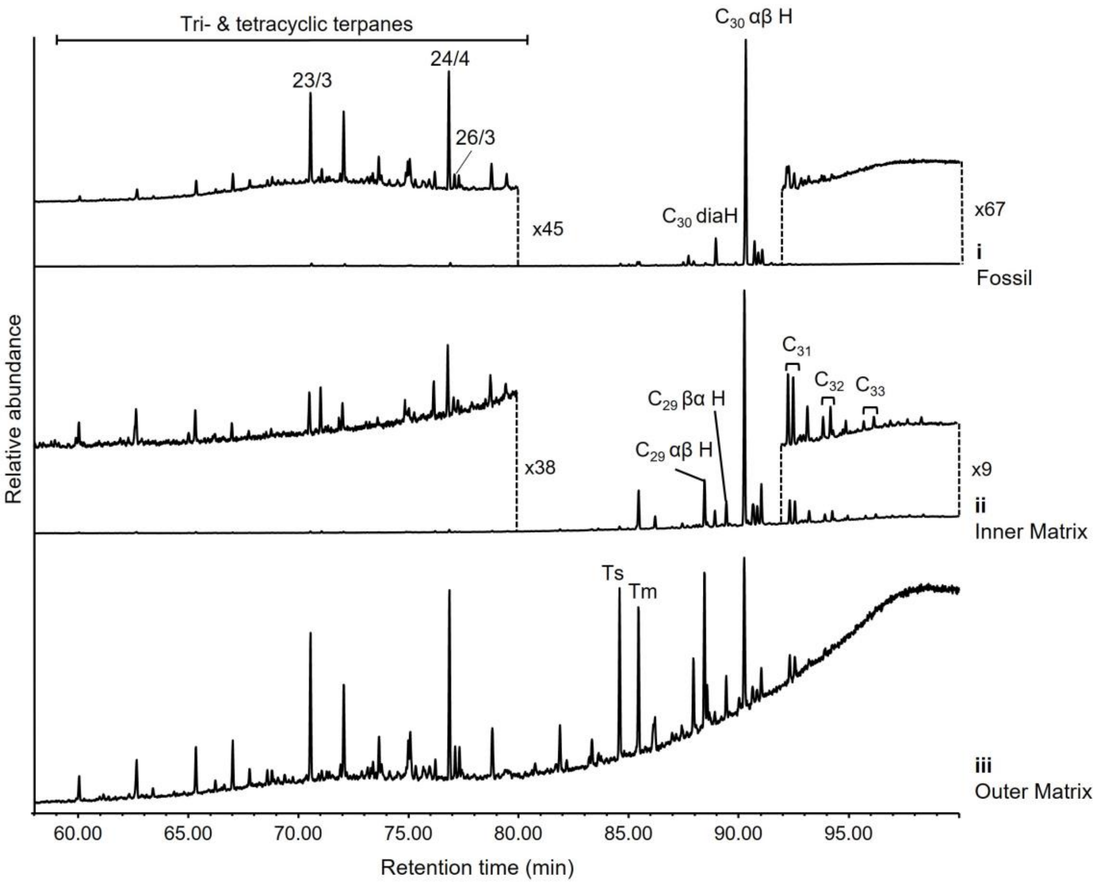

Figure S2. Extracted ion chromatograms *m/z* 191 of true fern sample TF-1 (*Diplazites unita*) (**i**) fossil; (**ii**) inner matrix; (**iii**) outer matrix. H: hopane; diaH: diahopane; 23/3: C_{23} tricyclic terpane; 24/4: C_{24} tetracyclic terpane; 26/3: C_{26} tricyclic terpane; C_{31}-C_{33} refer to regular hopanes (S and R isomers).

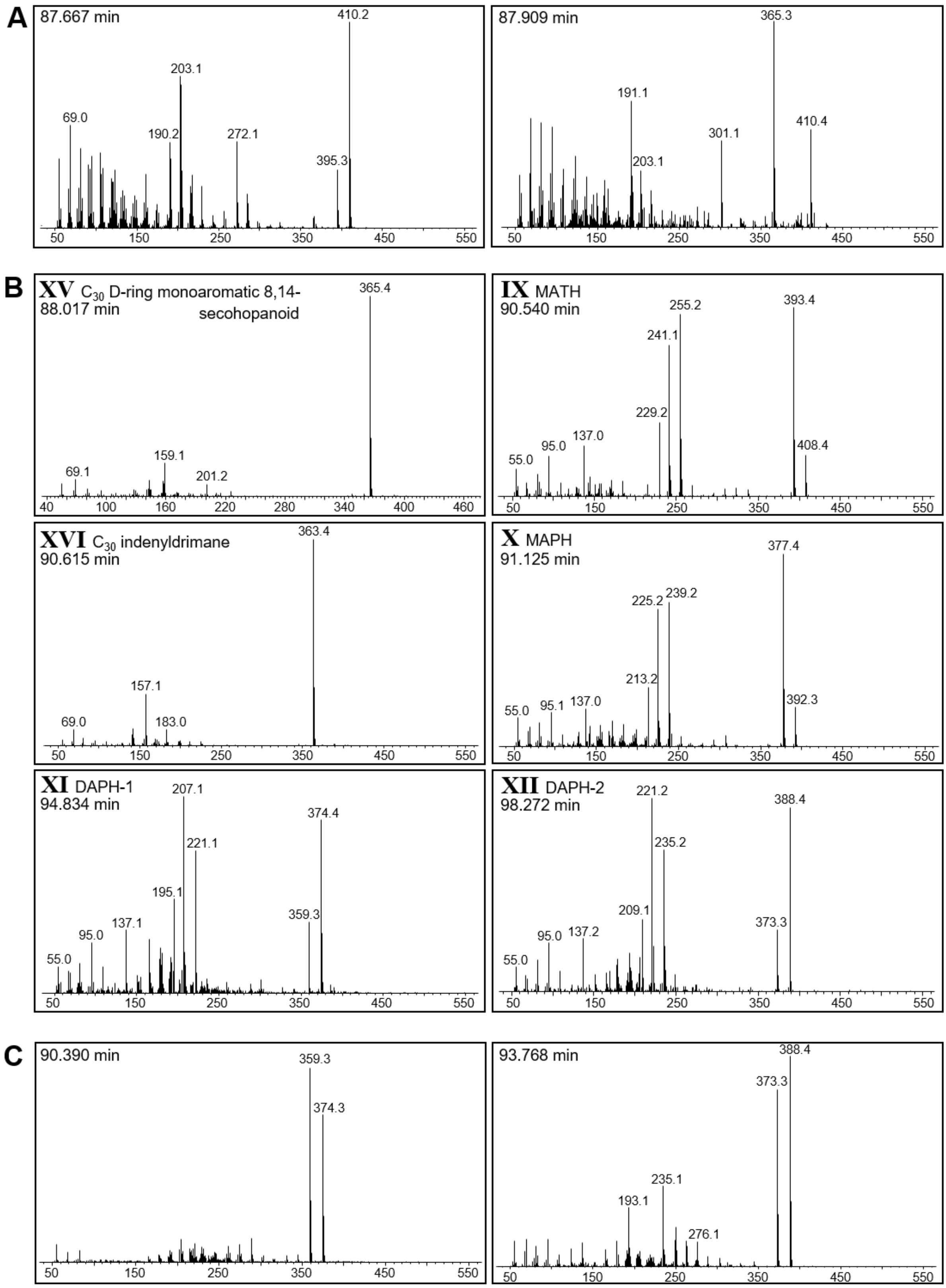

Figure S3. Mass spectra of various peaks identified in chromatograms from the true fern TF-1 fossil region; **A:** unidentified triterpenes in the saturated fraction (Fig. 2B); **B:** aromatised

fernane/arborane compounds; **C:** select unidentified aromatic compounds with similar mass spectra to a DAPH (**XI, XII**) structure (denoted by squares in Fig. 3A).

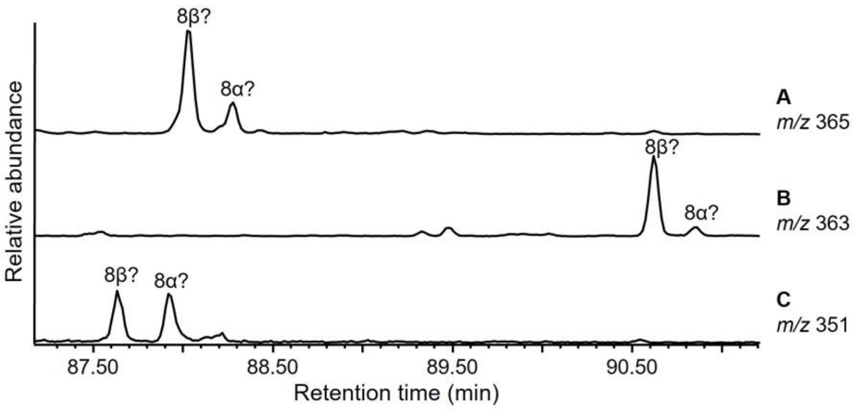

Figure S4. A: GC-MS *m/z* 365 extracted ion chromatogram showing C_{30} D-ring monoaromatic *seco*hopanoids; **B:** GC-MS *m/z* 363 extracted ion chromatogram showing C_{30} indenyldrimanes; **C:** GC-MS *m/z* 351 extracted ion chromatogram showing desmethyl D-ring monoaromatic *seco*hopanoids. All components are present as 8β- and 8α-isomers, whereby the 8β isomer dominates in D-ring monoaromatic *seco*hopanoids and indenyldrimanes, whereas an equilibrium between the C8-isomers is attained in the desmethylterpenoids. α- and β-nomenclature refers to stereochemistry of hydrogen at C-8. Compounds have been tentatively identified as the 8β and 8α isomers based on comparison with reported literature[1,2].

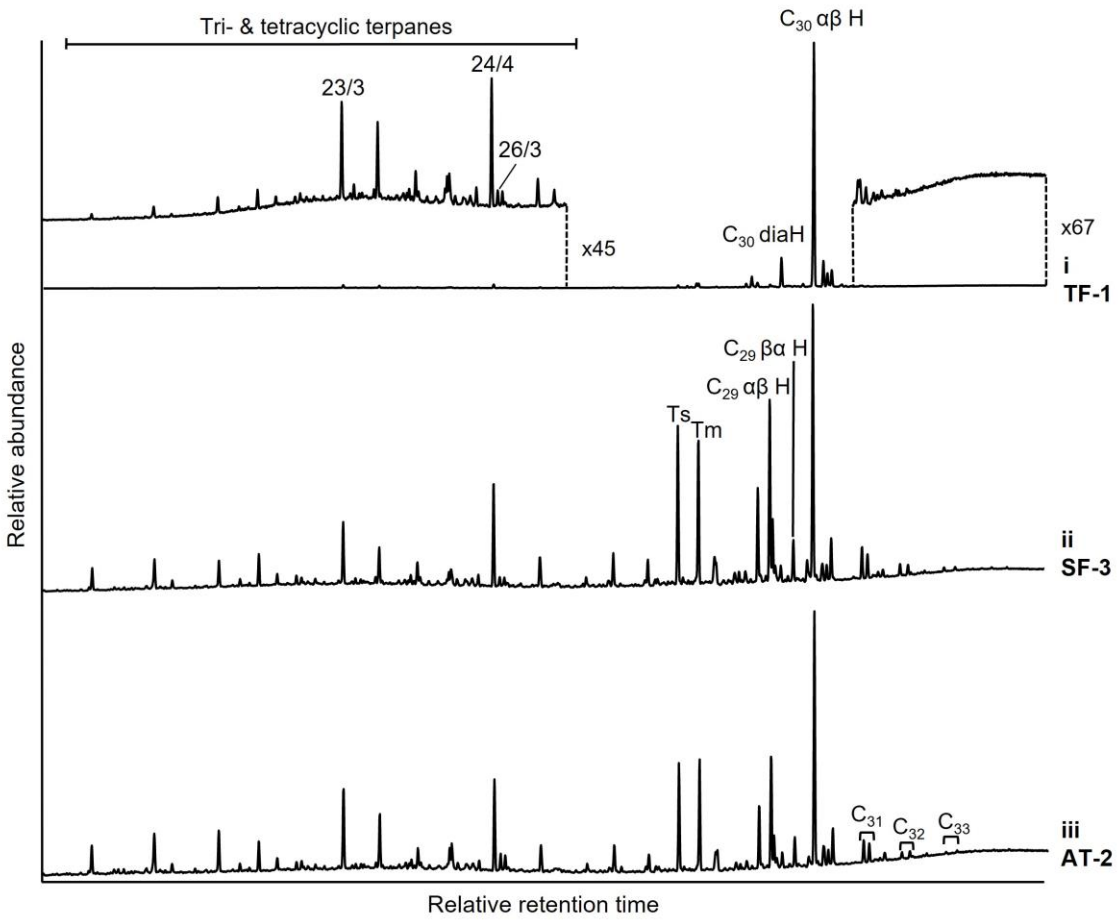

Figure S5. GC-MS extracted ion chromatograms *m/z* 191 of; **(i)** *Diplazites Unita* TF-1; **(ii)** *Odontopteris* sp. SF-3; **(iii)** *Asterophyllites* sp. AT-2. H: hopane; diaH: diahopane; 23/3: C_{23} tricyclic terpane; 24/4: C_{24} tetracyclic terpane; 26/3: C_{26} tricyclic terpane; C_{31}-C_{33} refer to regular hopanes (S and R isomers).

References

1. Niu, C. *et al.* Origin and Geochemical Implications of Hopanoids in Saline Lacustrine Crude Oils from Huanghekou East Sag and Laizhouwan Northeastern Sag, Bohai Bay Basin. *ACS Omega* **6**, 30298–30314 (2021).

2. Nytoft, H. P. *et al.* Biomarkers of Oligocene lacustrine source rocks, Beibuwan-Song Hong basin junction, offshore northern Vietnam. *Mar. Petr. Geol.* **114**, 104196 (2020).
